# Supplementary material for: Genetic control of rhizosheath formation in pearl millet
Source: Sci Rep. 2022 Jun 2;12:9205. doi: 10.1038/s41598-022-13234-w (PMC9163325; doi:10.1038/s41598-022-13234-w)
Supplement: Supplementary file 1 — Supplementary Information. [file 41598_2022_13234_MOESM1_ESM.pdf]

Figure S1. Association between cross-entropy (y axis) and number of ancestral populations, *K* (x axis). Each dot represents the smallest cross-entropy reached in ten runs using the sNMF algorithm (Frichot et al., 2014) from K=1 to K=10.

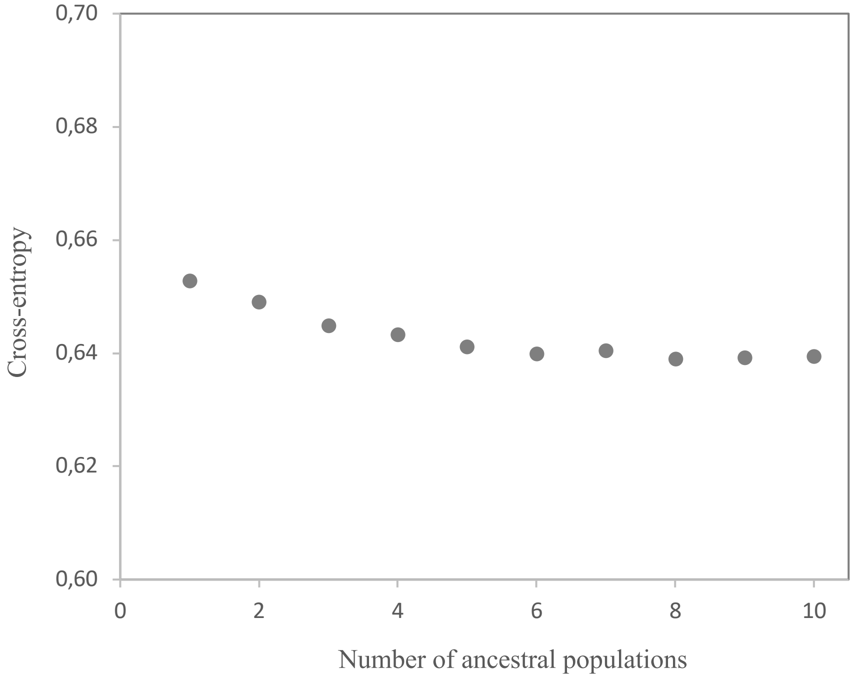

Figure S2. Frequency distribution of the root soil aggregation phenotype (i.e. RAS/RT) among the F2s and the contrasted inbred lines used in the development of the bi-parental population for Bulk Segregant Analysis.

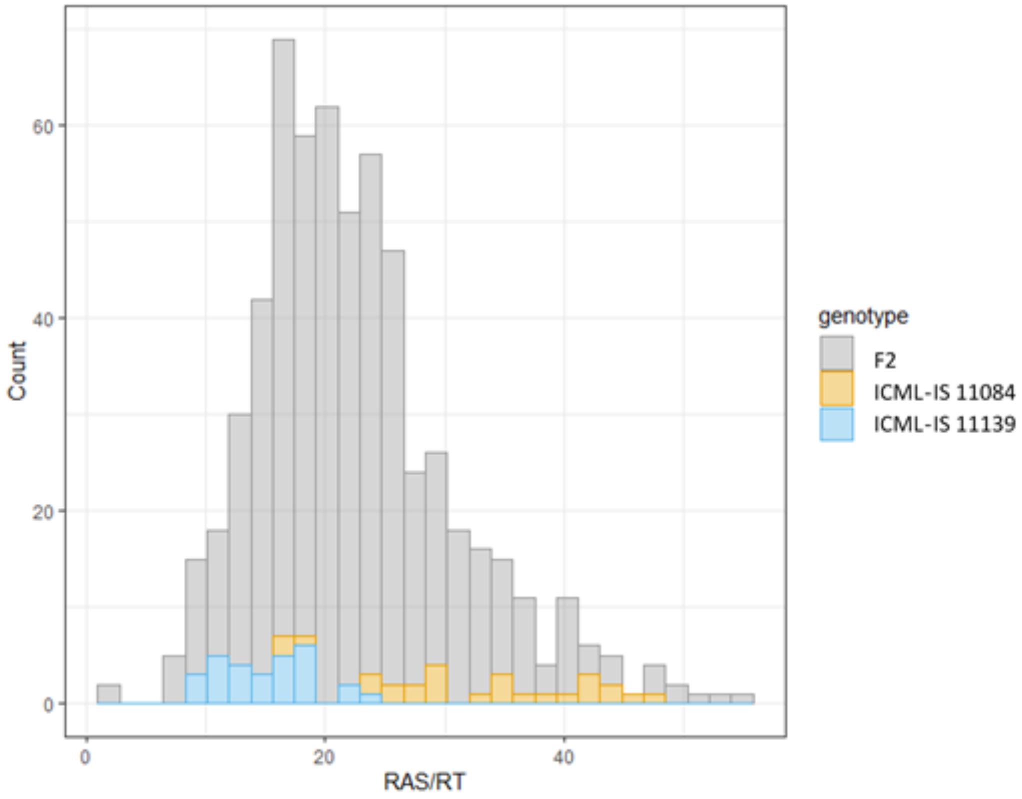

Supplementary Table S1. Spearman correlation between the different traits in 2018 and 2020 experiments

	Root architecture								Root hairs		AM symbiosis	
Trait	RAS/RT	L	RSA	AvgDiam	LFR	LTR	SAFR	SATR	AvgLRH	AgDRH	F%	I%
Combined experiments (2018 & 2020)												
RAS/RT	1	0,017	-0,081	-0,239*	0,053	-0,112	0,037	-0,110	0,278*	0,069	0,283	-0,133
L		1	0,908***	-0,155	0,986***	0,831***	0,978***	0,841***	0,068	0,074	-0,041	0,003
RSA			1	0,106	0,859***	0,878***	0,890***	0,896***	-0,099	0,185	0,109	0,194
AvgDiam				1	-0,278***	0,285***	-0,161	0,297***	-0,661***	0,545***	0,267	0,400
LFR					1	0,736***	0,979***	0,749***	0,150	0,031	-0,118	-0,085
LTR						1	0,771***	0,987***	-0,208*	0,196*	0,146	0,227
SAFR							1	0,786***	0,075	0,131	0,028	0,092
SATR								1	-0,229*	0,220*	0,176	0,269*
AvgLRH									1	-0,330***	-0,074	-0,023
AgDRH										1	-0,067	-0,467
F%											1	0,904***
I%												1
2018 experiment												
RAS/RT	1	0,174	0,018	-0,314**	0,225	-0,025	0,193	-0,013	0,293*	0,182	0,517	0,033
L		1	0,888***	-0,453***	0,988***	0,815***	0,978***	0,830***	0,311**	-0,063	0,180	0,319
RSA			1	-0,187	0,852***	0,824***	0,861***	0,852***	0,193	-0,017	0,140	0,336
AvgDiam				1	-0,546***	0,002	-0,491***	0,009	-0,298*	0,047	-0,061	-0,110
LFR					1	0,727***	0,986***	0,749***	0,346**	-0,014	0,208	0,338
LTR						1	0,724***	0,981***	0,095	-0,165	-0,110	0,038
SAFR							1	0,744***	0,357**	-0,018	0,229	0,372*
SATR								1	0,103	-0,153	-0,081	0,150

**AvgLRH**									**1**	**0,354***	0,550	0,133
**AgDRH**										**1**	-0,250	-0,383
**F%**											**1**	**0,727*****
**I%**												**1**
**2020 experiment**												
RAS/RT	**1**	-0,297	-0,308	**-0,341***	-0,300	-0,303	-0,305	**-0,322***	**0,410***	0,001	-0,165	-0,096
L		**1**	**0,992*****	**0,455****	**0,994*****	**0,944*****	**0,984*****	**0,952*****	0,085	-0,254	0,210	0,213
RSA			**1**	**0,520*****	**0,977*****	**0,966*****	**0,967*****	**0,974*****	0,053	-0,248	0,181	0,193
AvgDiam				**1**	**0,411****	**0,605*****	**0,409****	**0,623*****	**-0,338***	-0,002	-0,069	-0,072
LFR					**1**	**0,912*****	**0,994*****	**0,925*****	0,096	-0,267	0,218	0,213
LTR						**1**	**0,893*****	**0,993*****	0,019	-0,232	0,143	0,172
SAFR							**1**	**0,908*****	0,122	-0,262	0,168	0,165
SATR								**1**	0,004	-0,238	0,187	0,210
AvgLRH									**1**	-0,151	-0,074	-0,023
AgDRH										**1**	**-0,383***	**-0,385***
F%											**1**	**0,971*****
I%												**1**

Ratio (RAS/RT) between the mass of root-adhering soil (RAS) and root tissue biomass (RT), Total root length (L), Root Surface Area (RSA), Average Root Diameter (AvgDiam), Total Length of Fine Roots (LFR), Total Length of Thick Roots (LTR), Surface Area of Fine Roots (SAFR), Surface Area of Thick Roots (SATR), Average Length of Root Hairs (AvgLRH), Average Density of Root Hairs (AgDRH), Frequency of mycorrhization (F%), Intensity of mycorrhization (I%).

Table S2. **Significant marker-trait associations for root-adhering soil aggregation using 3 GWAS methods**

QTL No.	Chr.	Position (Mbp)	Minor Allele Frequency (MAF)	GWAS-LFMM $-log_{10}(pval)$	GWAS-MLM $-log_{10}(pval)$	GWAS-EMMA $-log_{10}(pval)$	PVE (%)	BSA sig region (95%CI)
1.1	1	56.7034	0.2158	4.0214			10.7	
1.2	1	66.9161	0.0504	4.1725		4.0898	10.7	
2.1	2	3.2903	0.0863	5.6404	4.6459	5.4942	14.7	
2.2	2	14.8369	0.3813	4.4356		4.3329	11.5	
2.3	2	19.1922	0.0791	4.9008	4.2480	4.7415	12.4	
	2	19.1934	0.0863	4.5321		4.4022		
	2	19.1935	0.0863	4.5329		4.3998		
	2	19.1944	0.0863	4.5321		4.4022		
	2	19.1945	0.0719	4.8858	4.2321	4.7278		
	2	19.2378	0.1007	4.2762		4.2181		
2.4	2	19.3640	0.1727	4.2129		4.2122	12.2	
	2	19.3706	0.0863	4.7196	4.1733	4.6267		
	2	19.3706	0.0863	4.7196	4.1733	4.6267		
2.5	2	22.8610	0.4101	4.2997		4.2163	11.0	
2.6	2	24.8134	0.1079	4.4889		4.2992	11.4	
2.7	2	61.8653	0.2086	4.7511	4.2907	4.8591	12.9	
2.8	2	81.9713	0.2302	4.3013			11.4	
2.9	2	90.4621	0.1655	4.0889		4.1451	10.9	
2.10	2	90.6436	0.2014	4.4009		4.1748	11.1	
2.11	2	199.3915	0.3381	4.2234		4.0852	11.7	
	2	199.3915	0.3381	4.5478		4.3925		
	2	199.3922	0.2806	4.3709		4.3693		
2.12	2	199.9019	0.3957	5.1952	4.5143	5.2611	14.0	
2.13	2	202.4478	0.4173	4.1268			14.9	
	2	202.5213	0.2806	5.7027	4.8508	5.5285		
2.14	2	211.3629	0.3525	4.2101		4.3523	11.6	
3.1	3	218.2121	0.1942	4.0622		4.2395	10.6	
3.2	3	252.1211	0.2086	4.0523			10.0	
4.1	4	54.2345	0.1223	4.7767	4.2154	4.9033	13.2	
	4	54.2347	0.1439	4.0398		4.2154		
	4	54.2347	0.1439	4.0398		4.2154		
	4	54.2348	0.1439	4.0398		4.2154		
	4	54.2349	0.1367	4.8568	4.3063	4.9683		
**5.1**	**5**	**3.2827**	**0.0504**	**4.1757**		**4.3339**	11.4	***RAS5.1***
5.2	5	32.0345	0.0863	4.4202	4.0362	4.4508	11.7	
**5.3**	**5**	**100.3439**	**0.1079**	**4.3818**			11.4	***RAS5.2***
5.4	5	138.7377	0.0360	4.0260			9.2	
**5.5**	**5**	**156.1388**	**0.1942**	**4.8798**	**4.3391**	**4.9033**	13.1	***RAS5.3***
**5.6**	**5**	**156.4967**	**0.2590**	**5.8391**	**5.0600**		15.6	
6.1	6	12.5248	0.2302	4.1207			9.7	
6.2	6	29.4980	0.1727	4.1224		4.2219	15.1	
	6	29.4980	0.1511	6.0203	4.9626	5.6540		
**6.3**	**6**	**227.6162**	**0.3453**	**4.3747**			11.2	***RAS6.2***
7.1	7	20.7928	0.1223	4.1928		4.1272	10.9	
7.2	7	31.2806	0.1223	4.9212	4.3726	4.9990	13.3	
7.3	7	60.0075	0.0863	4.0644		4.2022	11.2	
7.4	7	108.8972	0.0504	5.4493	4.7352	5.3799	14.4	
7.5	7	148.5773	0.0647	5.5623	4.7167		14.4	
	7	148.5784	0.0719	4.0643				
	7	148.6031	0.0719	4.8077	4.1811			
	7	148.6257	0.0719	4.0132				
	7	148.6516	0.0647	4.5648	4.0334			
7.6	7	149.8244	0.1655	4.9262		4.3235	10.5	

Significant markers (*p*-value <10^{-4}) identified by Latent Factor Mixed Model (LFMM) using the ridge algorithm (Caye *et al.,* 2019). The significant value of the statistic is shown for LFMM and two complementary GWAS methods: MLM and EMMA. The proportion of phenotypic variance explained (PVE) is shown for the most significant SNP within each QTL region. Shaded cells group the SNPs within the same QTL regions. In bold, significant SNPs coincidence with significant genomic regions identified in BSA.

Table S3. Root-adhering soil aggregation in the contrasted bulks of F2 lines (small *vs* high RAS/RT bulk) selected for the BSA study and the parental inbred lines of the cross.

	Bulk S-RAS	Bulk H-RAS	ICML-IS 11139	ICML-IS 11084
Number of individuals	55	55	29	27
RAS/RT Mean ± SD	10.99 ± 2.96	38.19 ± 6.42	15.01 ± 3.97	32.74 ± 9.08
RAS/RT Min – Max	1.64 – 17.13	28.91 – 54.76	8.44 – 23.28	15.76 – 47.41

Table S4. Top ten GO enriched terms in the differentially expressed genes between contrasting lines for root-adhering soil aggregation. Annotated: number of genes annotated with the corresponding GO term in the pearl millet genome; Significant: number of genes annotated in the differentially expressed genes with the corresponding GO term; Expected: number of corresponding GO term expected in the differentially expressed genes; Rank: rank of *p*-value in the classic Fisher test; *p*-value: *p*-value of the classic Fischer test; Weight01: weight01 in the Fischer test taking into account hierarchical links between GO terms.

GO term	Annotation	Annotated	Significant	Expected	Rank	p-value	Weight01
GO:0043531	ADP binding	357	70	16.33	1	9.00E-26	9.00E-26
GO:0016706	oxidoreductase activity	128	16	5.86	12	0.00024	0.00024
GO:0004601	peroxidase activity	197	20	9.01	16	0.00072	0.00114
GO:0020037	heme binding	557	41	25.49	20	0.00185	0.00185
GO:0019239	deaminase activity	9	3	0.41	23	0.00651	0.00651
GO:0016765	transferase activity	46	7	2.1	22	0.0046	0.00935
GO:0030598	rRNA N-glycosylase activity	20	4	0.92	27	0.01172	0.01172
GO:0003950	NAD+ ADP-ribosyltransferase activity	11	3	0.5	29	0.01194	0.01194
GO:0047134	protein-disulfide reductase activity	12	3	0.55	32	0.01539	0.01539
GO:0009045	xylose isomerase activity	5	2	0.23	33	0.01906	0.01906
